# Editorial: Modeling Play in Early Infant Development

**DOI:** 10.3389/fnbot.2020.00050

**Published:** 2020-08-06

**Authors:** Patricia Shaw, Mark Lee, Qiang Shen, Kathy Hirsh-Pasek, Karen E. Adolph, Pierre-Yves Oudeyer, Jill Popp

**Affiliations:** ^1^Intelligent Robotics, Department of Computer Science, Aberystwyth University, Aberystwyth, United Kingdom; ^2^Psychology, Temple University, Philadelphia, PA, United States; ^3^Infant Action Lab, Psychology Department, New York University, New York, NY, United States; ^4^Institut National de Recherche en Informatique et en Automatique (INRIA), Rocquencourt, France; ^5^The Lego Foundation, Billund, Denmark

**Keywords:** play, computational modeling, development, infant, learning

## 1. Introduction

This Frontiers Research Topic focuses on the question: Can we develop computers or robots that play and develop like children? Approaches to this question involves the elaboration and study of computational models of infant play with the perspective of two complementary disciplines. Firstly, developmental psychology benefits from such models to formulate theories and conjectures of infant play which can be tested and evaluated through experimental studies. Secondly, the new field of developmental robotics looks toward infant development for inspiration, data, and guidance, in order to build models of learning that may be useful both for better understanding of human development and for engineering autonomous learning in robots and other systems.

These fields have common ground in this very active and significant research area, investigating how babies learn and grow cognitively, and testing our knowledge in the concrete world of computer models. A major characteristic of early human development is the open-ended acquisition of new abilities and competencies. Human infants are born helpless yet they actively become familiar with their environment and their own body through spontaneous exploration and interaction with others. Within a few months of rapid learning and development, they have acquired quite sophisticated sensory-motor and social competences. New skills appear to sprout from current competences as experience builds along a continuous trajectory of action and interaction. In particular, such open-ended learning is readily seen in the ubiquitous behavior known as play.

Play can be used to describe an expansive range of exploratory activities, but the concept currently lacks a sufficiently unifying theoretical framework. Here we focus on forms of play which involve free and spontaneous intrinsically motivated exploration of actions, objects, places, or tasks and activities in varying contexts, outside motivation to fulfill basic physiological needs like feeding and without external goals set by social peers. Such forms of exploration may involve the search for novelty or surprise, can be goal-free but also involve self-generated goals which are pursued for their intrinsic “interestingness.” For example, when encountering novel objects or events, infants will often display pleasure in the interaction, try to repeat the experience and show enjoyment of their own activity. This suggests an enactive approach which Jerome Bruner called “learning by doing.” Von Hofsten describes play as “the purposeful seeking of enjoyable action possibilities,” and Vicky Bruce stresses the immersive aspects in terms of several features of “free-flow” play.

From developmental robotics, work on these ideas have explored both solitary play with objects and early interactive play with others as a generative behavior that combines fragments of past experience with new sensory-motor events in differing contexts. Computational models of play have been proposed, for example based on forms of novelty or information gain as an intrinsic driver, leading to designs for investigations on “curious robots.”

The aim of this Frontiers Research Topic is to present international state-of-the-art research from naturalistic or experimental infant studies and computational/robot modeling, on early infant play behavior. The focus will be on the very earliest forms of play, because this is concurrent with increasing perception and understanding of the “physics of the world,” e.g., perceptions of objects, causality, and interactions. Many interesting questions arise: for example, how does play emerge and what is its relation to goal-free motor babbling? How does play relate to object understanding and world knowledge. How does intrinsically motivated self-generation of goals relate to future extrinsically motivated goal generation and goal attribution? How far can the world be explored through the paradigm of play? How can we best understand more about infant cognition from modeling these concepts on robots? This topic includes leading contributions delivering experience and original research on computational modeling of psychological experiments about these topics, as well as experimental and theoretical papers that increase understanding of these important issues and core concepts in infants and machines.

## 2. Overview of the Contents of the E-Book

The papers in this Research Topic are broken down into three categories. Firstly there are studies from Developmental Psychology of infants whilst playing, to help define the broad spectrum of play. Then we have the theoretical models exploring different aspects of this observed play behavior, before finally moving onto the application of models for playful learning to robotics to learn how to perform various tasks. Below is a summary of the various papers in this Research Topic.

### 2.1. From Developmental Psychology

Whilst previous attempts have tried to give a single definition for all types of play, or are restricted to the concept of free-play. Zosh et al. provides a new definition that describes play as a continuum from free-play through to directed-play. The level of engagement or direction from adults increases as you move along the continuum, allowing this new definition to better represent and review the importance of these different types of play.

At it's core, the foundation of definitions in play stem from the work of Vygotsky (1967) and Piaget (1952). These are all expanded here where working definitions and literature reviews are given for the common characteristics of play being; Active, engaged, meaningful, social, iterative, and joyful. Overall this gives a multi-dimensional space in which different types of play can be defined, opening up avenues for future research.

#### 2.1.1. Social Interaction for Play

Related to this, Cochet and Guidetti reviews two decades of research into Joint Actions and the importance of the social element for Human Robot Interaction (HRI). As part of the review, they focus on the development of joint attention through play for infants, breaking down the interaction based on three dimensions: motor precision, coordination, and anticipatory planning. By considering each dimension in isolation, they aim to support developmental roboticists in the modeling and learning of the behavior, whilst also providing developmental psychologists a platform on which to disentangle these and assess the “manipulability” of each dimension individually.

The dimension of motor precision requires not only the robot understanding its own motor skills, but also the kinematics of the human participant, e.g., reachability or graspability of an object. The use of gaze and pointing are also key elements here for identifying the object or event on which the joint attention is based. Note that to support the involvement of the human, the gaze should shift between the human and the target.

The second dimension of coordination considers the synchronization of behaviors as well as the multi-modal communicative signals (gaze, gesture, vocalizations, facial expressions, etc.), and use these to adjust the robot's own behavior.

Finally, the dimension of anticipatory planning considers the individuals ability to predict the behaviors of the partner as part of a sequence of actions in order to enable better coordination and anticipatory behaviors in support of the other person. The need for inner states such as those representing the beliefs of the partners is still an area for debate, but what is clear is the need for quick responses (in the order of 100 ms) in order to maintain the feeling of effective interaction.

Overall, this review provides a roadmap toward enabling robots using human-like communicative modalities to invite more natural interactive behaviors with people.

#### 2.1.2. Guided Play

Meanwhile, Yu et al. specifically focuses on guided play, providing a perspective on the existing literature and how this could be used to both form theoretical models for both studying this type of play in humans as well as developing models for robots.

Through the observations of effective methods adapted to individual learners, data analytic approaches could be applied to more accurately predict the current state of the learner and the effectiveness of the guidance, as well as starting to suggest and improve the ability of automated tutors. The important feature of any model is the need to be dynamic, and adapt to the interactions over time for each individual. Building on social cues such as gaze direction, a more naturalistic interaction can be achieved leading to better engagement and learning.

Gliga, explores the literature on the importance of variability in behavior for promoting learning, specifically considering motor acts for reaching, locomotion, and vocal behaviors in a variety of species. Through considering differences in the types and variability of motor actions between normally developing infants and those with various conditions leading to atypical development (e.g., Cerebral palsy or brain damage), they identify the importance of certain types of variability in motor actions that support development and learning.

They start by differentiating between planned noise, variability generated in the central nervous system and execution noise, variability resulting from the randomness of biological processes. These are then classified into three main sources of variability present during infancy; Hypothesis testing, Learning expectant variability and Sensory-motor noise. It is clear that the first two are directly linked to learning, but the third still needs further fine grained investigation. Studies such as those by Thurman and Corbetta can start to investigate some of the finer details, whilst Chastain considers a more evolutionary change in phenotypic variation demonstrated through motor babbling by re-evaluating the “Baldwin Effect.”

Neale et al., investigates the potential for more fine grained analysis of play behavior. In order to truly develop a multi-model definition of play behavior, we would need to combine behavioral, cognitive and neurological measures together. Currently, most measures of behavior and cognition are very coarsely grained, i.e., 10 s of seconds, hours, days, weeks, or months, whilst the neurological measures are in milliseconds (ms). If there is to be any hope of aligning them, all need to be measurable on the same scale. Neale et al., develops a framework for measuring sensorimotor, cognitive and socio-emotional play in the ms timescale for future alignment with EEG recordings, building on interdisciplinary studies of play behavior by Miller (2017). Observing adult-infant interactions during play and non-play conditions, a precise coding system was defined for each of the three measures and applied in 33 ms intervals (30 fps). Combining the three measures, a clear separation is visible between the play and non-play behaviors with further sub-coding in each measure enabling finer grained evaluation. Whilst incorporation of the EEG data collected during the study is left to future work, this study concludes with a summary of how the potential analysis could be done.

In the study by Markova, the relationship between play and the hormone oxytocin was evaluated. The hormone, sometimes referred to as the “cuddle hormone,” is recognized as supporting cooperation in adults. Mothers with infants aged 4-months engaged in a period of natural play.

The types of play considered were highly structured involving both verbal and non-verbal communication, where the non-verbal was in the form of facial expressions and gestures. For early infants, it was previously unclear how much they responded to disruption in this structured play, e.g., missing actions from a song.

By taking various swab samples before and after some structure natural play, they were able to identify a strong correlation to engagement with play, indication that social games are an important part of early mother-infant interaction.

#### 2.1.3. Longitudinal Studies

Thurman and Corbetta review data from one of their previous longitudinal studies to consider the postural changes between mothers and infants as early infants develop from sitting to walking, and how these postural changes are linked to exploratory behaviors on objects. Specifically, they ask the questions; do infants and mothers alike shift interactive behaviors as infants acquire locomotion? Do interactive behaviors depend on the posture performed in the moment? And, do transitions between targets occur while maintaining or changing posture?

Analysis of postures used predefined techniques (Touwen, 1976), to coarsely classify postures such as sitting, kneeling, crawling, or standing. The types of interaction were classified as targeted/untargeted, interaction, passive, fine motor, or gross motor.

Observing infants every 2 weeks during 10 min of free play starting from 6 months old up to five sessions after the onset of walking they observed significant and increasingly varied use of the full body to explore and interact with their environment. Throughout this developmental period, mothers produced little to none or purely passive activity during the sitting, kneeling/squatting, and standing phases.

In another longitudinal study by Muentener et al., five different measures for play were evaluated in relation to cognitive development over a 9 month period. These measures included attention to novelty, inductive generalizations, face preference, imitative learning, and efficiency of exploration.

Infants aged 5–19 months were observed 4 times over a 9 month period during 15 min sessions of exploratory play, with a variety of objects provided by the tester related to each of the measures being investigated. A later assessment on a subset of the individuals was done at 3 years old, assessing vocabulary size and IQ.

Over the range of measures considered, efficiency of exploration correlated with higher IQ scores at the final assessment.

Tian et al. perform a cross-sectional study of pre-school children in a block-building task. Variables in the methodology include the group size (1, 5, or 10), the form in which the model was presented (3D model vs. 2D pictures) and the age of the participants (K1–K3) in a public kindergarten. The measures of the task were then broken down to consider three different skills relevant to the block-building task (block building, structural balance and structural features) as well as considering the variation between genders alongside the other variables in task performance.

Significant variation from gender was identified in each of the block-building categories except structural features. Block-building skills improved across the age dimension, and the 3D model was found to elicit more representational play than the 2D pictures. Finally the small group size performed slightly better than the individuals or large groups, possibly due to interference when group size was too large.

### 2.2. Modeling Play in Infants

The contributions described above each include direct observations of infant behavior in various situations. In many of these, the details provided an outline for models to be constructed and then compared against. The following contributions each focus on different aspects and approaches to starting to model the observations by the Developmental Psychologists.

#### 2.2.1. Theoretic Models

Chastain presents an information theoretic approach to modeling learning, building on previous theories by Baldwin (1902). They discuss the many divergent interpretations of the Baldwin Effect for evolutionary theory and attempt to bring back interpretations from the original work, bring it back toward Developmental Psychology and specifically related to the role of abstraction in phenotypes. These theories consider evolution and development of complex skills over generations based on phenotypic plasticity. This allows organisms to try out motor actions to obtain reward signals in their juvenile state and enables motor babbling as a learning mechanism to smooth the fitness landscape. This can be observed in skills development such as hand writing where a level of imitation from one generation to the next speeds up the learning process and development of these complex skills.

Schank et al. also takes the approach of developing a theoretic approach to modeling learning, this time using a game theoretic approach to demonstrate how fair play in juvenile animals can lead to fair behavior in adults. Fair play is often observes in many species (Burghardt, 2005), with behaviors such as self-handicapping (e.g., an individual not biting as hard as it can) and role-reversal (e.g., alternately switching between dominant and submissive positions). In adults, the fair behavior can be observed as the social group sharing food instead of hoarding all the food for an individual. By modeling a “play” gene that is either on or off, and two stages of development (juvenile and adult), they evaluate the activation of the play gene in animals, incorporating a “gestation” period between reproduction cycles. When compared against control simulations, they found that the play gene evolved to be activated significantly more across a wide range of conditions. This supports the argument that one of the benefits to play is for learning social skills and to facilitate the acquisition of skills for behaving fairly as adults.

#### 2.2.2. Robotic Modeling

Mannella et al. investigate the application of Competence-based Intrinsic Motivation (CB-IM) for driving the discovery of goals, and maintaining focus for learning a behavior until a goal is satisfied. This approach to driving learning is applied to learning a body model and kinematics of a 6DoF robot arm, through self touch on a simulated robot arm with touch sensors evenly distributed. The model combines a neuro-inspired RNN (Mannella and Baldassarre, 2015), with a random trajectory generator and an associate memory. The “easy” to reach contact points are learnt first before gradually building up the complexity of goals to the more challenging configurations, refining the reach actions. The contribution finishes by making three predictions based on the model for Developmental Psychology. These are related to the efficiency of reaching as infants develop as well as the reaching to points on the body related to the complexity of the reach and the uneven distribution of tactile receptors throughout the body.

Related to this, Kumar et al. also demonstrates modeling a schema based learning approach on a robotic platform, constructing increasingly complex actions through chains of simpler actions. Inspired by the ideas of Piaget (1952) and building on a schema based model by Sheldon and Lee (2011), the model is extended to enable hierarchical building of chains that can themselves become reusable unit actions, with both partial and complete generalization. Rather than being performed in simulation, in this case the learning is evaluated on-line on an iCub humanoid robot where the robot learns to grasp objects, and move a specific object to a key point to unlock a toy. The learning is performed online with new schemas and chains of schemas being constructed hierarchically. Properties of objects are considered to enable reuse of similar schemas and for generalization of schemas to reduce the overall number of schemas required. The experiments also consider individual variation in preferences between infants, rather than attempting to model the average results from observations. A set of preferences are defined with weightings to shift between them. Currently these weightings are static, but future work will consider how they may change based on the current situation, e.g., based on internal measures such as happiness or satiety.

Meanwhile, the ability to play football has long been a golden target for humanoid robotics. Ossmy et al. trained Nao robots based on toddler movement patterns to improve the ability of the robots to quickly navigate around the playing field. Simulated robots were trained on movement paths generated by toddlers, including stopping and starting movement intermittently, vs. robots trained on less varied geometrical paths. Games played between the two groups demonstrated that the increased variability of the movement patterns from human infants let to better performance in the matches. Not only does this paper show how robots can benefit from the observations of infant development, but also how robots can be used to test hypotheses about infant development.

Using the Nao humanoid robot as a basis for the simulation, and an existing system for training a walking system, MacAlpine et al. (2012), they focused on a used a reward based system to tune a set of the parameters for refining the walking system. When testing the robots trained on the infant patterns vs. those trained on more traditional geometric patterns, the infant trained robots consistently beat all those trained on different geometrical patterns. Further breaking down the infant patterns based on levels of exploration, the robots trained on the patterns showing the most exploration also went on to win the most games in a tournament. Overall, this emphasizes that variability is a feature of infant development, rather than a stumbling “bug” in the process.

This final contribution the topic by Wu et al. shows the benefit of developmental learning and play applied to another robot, this time a mobile robot that learns through stages to look at, reach and grasp, and move toward balls in its environment. Structured as a game with a simple and complex mode, the study uses the concept of Lift-Constraint, Act, and Saturate (LCAS) (Lee et al., 2007), to aid the robot learning the stages of the game to ultimately succeed in the complex game requiring the robot to drive around to visually locate the balls and pick them up. The grasping of balls requires the coordination of “hand-eye” movements that are also learnt in a stages approach. The model is implemented through the use of a Radial Basis Function (RBF) network that is trained using data collected by the robot. The training samples used are limited by the constraints applied based on the current stage of development. A comparison of learning without the constraints shows that the constraints enable to the robot to learn faster.

## 3. Conclusion

Here we have brought together studies that help to further define the broad spectrum of play based on infant studies, formations of theoretical models based on these definitions, and applied models to robotics platforms for developmental inspired learning approaches. Of course the process does not stop there, as the theoretical and robotic models will ultimately feedback to the Psychology to help better understand the behaviors observed.

The studies from Developmental Psychology provide a framework and roadmap for the implementation of theoretical and robotic models. Through the application of developmental stages, the studies here have demonstrated the gains to be made in improved final performance and rate of learning. Not only that, but they have also provided a test bed for the evaluation of hypotheses related to development in infants.

## Author Contributions

All authors acted as guest editors for the related Research Topic. This editorial was produced by PS.

## Conflict of Interest

The authors declare that the research was conducted in the absence of any commercial or financial relationships that could be construed as a potential conflict of interest.
